# ENKUR expression induced by chemically synthesized cinobufotalin suppresses malignant activities of hepatocellular carcinoma by modulating β-catenin/c-Jun/MYH9/USP7/c-Myc axis

**DOI:** 10.7150/ijbs.67476

**Published:** 2022-03-21

**Authors:** Rentao Hou, Yonghao Li, Xiaojun Luo, Wan Zhang, Huiling Yang, Yewei Zhang, Jiahao Liu, Shaohua Liu, Siyuan Han, Chen Liu, Yun Huang, Zhen Liu, Aimin Li, Weiyi Fang

**Affiliations:** 1 Cancer Center, Integrated Hospital of Traditional Chinese Medicine, Southern Medical University, Guangzhou 510315, China.; 2 Department of Medical Oncology, Guangzhou First People's Hospital, School of Medicine, South China University of Technology, Guangzhou 510180, China.; 3 School of Pharmacy, Guangdong Medical University, Dongguan, Guangdong 523808, P.R. China.; 4 Key Laboratory of Protein Modification and Degradation, School of Basic Medical Sciences, Affiliated Cancer Hospital and Institute of Guangzhou Medical University, 511436 Guangzhou, China.; 5 Department of General Surgery, Pingxiang People's Hospital, Pingxiang, Jiangxi 337000, China.; 6 Department of Oncology, SSL Central Hospital of Dongguan, Southern Medical University, Dongguan, Guangdong 523000, China.; 7 Department of geriatric medicine, The First Affiliated Hospital of Fujian Medical University, Fujian, Fuzhou 350004, China.

**Keywords:** Chemically synthesized cinobufotalin, Hepatocellular carcinoma, Sorafenib, Malignant activities

## Abstract

ENKUR plays a crucial role in lung and colorectal cancers. Chemically synthesized cinobufotalin (CB) showed its significant anti-cancer effect in nasopharyngeal carcinoma. However, the roles of ENKUR and CB along with their correlation are still unknown in hepatocellular carcinoma (HCC). In this study, ENKUR expression in HCC tissue and cells were detected. The relationship between ENKUR expression and clinical pathology was also assessed. *In vivo* and *in vitro* experiments were conducted to explore the effects and molecular basis of ENKUR and CB in HCC. ENKUR expression was correlated with HCC progression and patient prognosis. Furthermore, ENKUR could inhibit tumor proliferation, metastasis, and sorafenib resistance in HCC. Mechanistic studies showed that ENKUR or its Enkurin domain could bind to MYH9 and decrease its expression by binding to β-catenin and inhibiting its nuclear transfer, thus decreasing c-Jun level. Low expression of MYH9 suppressed recruitment of deubiquitination enzyme USP7, promoting degradation of the c-Myc. Therefore, cell cycle and EMT signals were suppressed. CB as a safe and effective anti-cancer compound up-regulates the expression of ENKUR via inhibiting PI3K/AKT/c-Jun-mediated transcription suppression. These findings show that ENKUR induced by CB antagonizes β-catenin/c-Jun/MYH9/USP7 pathway, thus increasing c-Myc ubiquitin degradation and finally suppressing cell cycle and EMT signals.

## Introduction

Hepatocellular carcinoma (HCC) is a highly malignant tumor with high morbidity and mortality worldwide 1,2. However, conventional HCC treatments, including surgical resection and transplantation, are only effective in patients diagnosed at early stages 3. HCC is characterized with abnormal expression of a lot of genes 4-6, which results in frequent occurrence and distant metastasis of tumor as well as poor prognosis of HCC patients 7,8. So far, there is no particularly effective therapeutic options for advanced HCC patients 9. Sorafenib is a first-line drug that only increases the median survival of HCC patients by three months 10. Moreover, most patients develop disease progression due to sorafenib resistance. Therefore, the molecular basis of HCC should be explored to discover a new treatment strategy.

ENKUR was first reported as an adaptor essential in the localization of a Ca^2+^-permeable ion channel in sperm 11. ENKUR has three potential protein-protein interaction domains (C-terminal region, a proline-rich N-terminal region that interacts with PI3K, and IQ motif that binds the Ca^2+^ sensor). Ma et al. recently reported that ENKUR suppresses tumor proliferation, migration, and invasiveness in colorectal cancer and lung adenocarcinoma 12,13. However, the role of ENKUR in HCC is unclear.

MYH9 encodes myosin IIA heavy chain involving in the pathogenesis of many tumors. In the initial study, MYH9 was reported as a tumor suppressor participating in the pathogenesis of squamous cell carcinoma 14,15. However, some reversed studies showed that MYH9 functions as a potential oncogene in most tumors and promotes the tumor occurrence, metastasis and tumor stemness formation 16-19, including esophageal squamous cell carcinoma and nasopharyngeal carcinoma 20,21. These studies show the complexity and importance of MYH9 in tumor pathogenesis. Interestingly, we also have confirmed that MYH9 significantly promotes cell tumor stemness, metastasis and chemoresistance by interacting with FOXO1, GSK3β or HBX in NPC and HCC 22,23. In subsequent study, Co-IP assays combined with Mass spectrometry analysis showed that ENKUR interacts with MYH9 in ENKUR-overexpressing A549 cells (data not published). However, the relationship between ENKUR and MYH9 in HCC is still unknown.

Natural cinobufotalin (CB) is a bufadienolide derived from toad venom and has anti-tumor activities in several cancers 22,23. Chemically synthesized CB can markedly suppress the malignant phenotypes of NPC, including tumor stemness^22^,^24^ and indicating its anti-cancer activities. However, the role of chemically synthesized CB in HCC pathogenesis and the combined effect of CB and sorafenib are unclear.

In this study, ENKUR inhibited sorafenib resistance and HCC development by binding to β-catenin in the HCC cytoplasm. Moreover, chemically synthesized CB induced ENKUR expression, suppressing HCC malignant activities by antagonizing β-catenin/c-Jun/MYH9/USP7 pathway and thus promoting c-Myc ubiquitin degradation.

## Materials and Methods

### Cell culture

LO2, HepG2, Hep3B, MHCC97H, and HCCLM3 cell lines were sourced from Shanghai Zhong Qiao Xin Zhou Biotechnology Co. Ltd. HEK293T cells were stored in the central laboratory of our hospital, Guangzhou, China. The cells were cultured in a DMEM medium containing 10% fetal bovine serum at 5% CO_2_ and 37 °C.

### Cell transfection

ENKUR and C-Jun plasmids were obtained from GeneChem (Shanghai, China) and Vigene Biosciences (Shangdong, China), respectively. siRNAs were purchased from Guangzhou RiboBio Co. Ltd (Guangzhou, China) (Supplementary Table 1). The siRNAs were transfected into cells using Lipofectamine TM 2000 (Invitrogen Biotechnology Co., Ltd., Shanghai, China). These cells were collected for further experiments after 48-72 hours of transfection.

### Lentivirus

Lentiviral particles with pGC-FU-ENKUR-GFP plasmids and control vectors were obtained from GeneChem (Shanghai, China). The lentiviral particles were then infected with HCC cells. qPCR and Western blot was used to detect ENKUR expression.

### PCR

Total RNAs were extracted from HCC and normal liver cells. The total RNAs were then reverse-transcribed into cDNA using reverse transcription reagents (TaKaRa Bio, Inc., Shiga, Japan) with specific primers (Supplementary Table 2). BIO-RAD T100 and BIO-RAD CFX 96 detection systems were used for RT-PCR and QPCR experiments, respectively.

### Western blot analysis

The cell lysates were extracted using a lysis buffer. Protein concentration was then determined using the BCA protein determination kit (Tiangen Biotechnology Co., Ltd., Beijing, China). The proteins were separated and transferred using SDS-PAGE and micromolecular membranes. Finally, enhanced chemiluminescence reagents (Thermo Scientific, Waltham, MA, USA) were used to visualize specific proteins. The following antibodies were used: Anti-Flag (F7425, Sigma, 1:1000), His-flag (8480, Proteintech, 1:1000), c-Jun (9165, CST, 1:1000), β-catenin (8480, Proteintech, 1:1000), E-cadherin (60335-1-Ig, Proteintech, 1:1000), N-cadherin (66219-1-Ig, Proteintech, 1:1000), Vimentin (10366-1-AP, Proteintech, 1:2000), β-actin (60008-1-Ig, Cowin, 1:2000), GAPDH (60004-1-Ig, Cowin, 1:2000). Images were obtained using MiniChemiTM Chemiluminescence Imaging System (Sagecreation, Beijing, China).

### Boyden and wound healing assay

For boyden (BD Biosciences, NJ, USA) assay, 100 µl cell suspension was inoculated into a boyden chamber coated or uncoated with Matrigel (BD Biosciences, NJ, USA). The bottom chamber was filled with an appropriate amount of medium containing serum. The cells were stained with crystal violet after migration. For wound healing assay, a sufficient amount of cells was seeded in a 6-well plate. A pipette tip was used to develop scratch for assessing the progress of the migration at the beginning and 48 hours after the scratch.

### MTT assay

The MTT assay was used to examine cell proliferation and drug sensitivity. Several cells were inoculated into the 96-well plate and incubated overnight to allow the cells to grow adherently. MTT reagent (5 mg/ml) (Sigma-Aldrich, MO, USA) was used to determine cell viability.

### EdU incorporation assay

Apollo567 *in vitro* Imaging Kit (RiboBio Co., Ltd, Guangzhou, China) was used for EdU incorporation assay, following the manufacturer's instructions. The cells were incubated with EDU reagent (10 µM) for 2 hours, fixed in 4% paraformaldehyde. The cells were labeled using Apollo fluorescent dye and 5 µg/ml DAPI after permeabilization using 0.3% Triton X-100.

### Immunofluorescence test

Appropriate amount of cells was inoculated in a 48-well plate (on a glass slide). Paraformaldehyde (4%) and Triton X-100 were used to fix and permeabilize cells, respectively. The cells were incubated with antibodies, then counterstained with 0.2 mg/ml DAPI for visualization using a fluorescent confocal microscope (Carl Zeiss LSM800).

### Subcutaneous xenograft mouse model construction

The subcutaneous xenograft mouse model was used to test tumor growth. The cells (100 µl, 5×10^6^) were subcutaneously inoculated into the flank of 3-4-week-old female mice (N=5 per group). The experiment was stopped on the 15th day after cell seeding, and then the tumor was removed and weighed. Tumor volume was determined using the formula: (length × width^2^ × 0.50). The subcutaneous xenograft mouse model was also used to evaluate the abilities of CB and sorafenib to suppress tumor growth. The cells (5×10^6^) were subcutaneously transplanted to the flanks of 3-4-week-old female BALB/c-nu mice. The mice were then divided into five groups (N=5 per group) after three days: Normal Saline (NS) group, 4 mg/kg CB group (CB4), Sorafenib group (S), 8 mg/kg CB group (CB8), and Sorafenib + 4 mg/kg CB group (S+CB4). The mice were orally given sorafenib (30 mg/kg) once daily or intraperitoneally administered with different concentrations of CB every three days for three weeks. Kaplan-Meier curves were used to examine the overall survival times.

### CB cytotoxicity assay

CB cytotoxicity was evaluated using liver and kidney function tests. Female BALB/c-nu mice (3-4-week-old) were divided into seven groups: phosphate buffer saline (PBS) group, 4 mg/kg CB group (CB4), 8 mg/kg CB group (CB8), 12 mg/kg CB group (CB12), 20 mg/kg CB group (CB20), sorafenib group (S), and sorafenib +4 mg/kg CB group (S+CB4). The mice were orally given sorafenib (30 mg/kg) once daily or intraperitoneally administered with different concentrations of CB every three days for three weeks. The mouse eyeballs were removed, blood obtained, then centrifuged to obtain serum. Serum Alanine aminotransferase (ALT), Aspartate aminotransferase (AST), ALP, ALB, UR, and urea levels were then determined.

### Metastasis assay

The mice (five mice per group) were injected with cell suspension (100 μl, 3×10^6^) via the tail vein. Nude mice were sacrificed after six weeks of feeding, then the lung tissues were obtained. OLYMPUS whole-body fluorescence imaging system (Olympus Corporation, Tokyo, JPN) was used to image the lung tissues.

### Luciferase reporter assay

The effect of c-Jun on the transcription activity of ENKUR was explored using luciferase reporter assays. Sequences of ENKUR promoter containing c-Jun binding sites and their control mutation sites were cloned to the pGL4.1-Basic luciferase reporter vector. Dual-Luciferase Reporter Assay System (Promega Corporation, Madison, WI, USA) was used to assess luciferase activity after the plasmids were co-transfected with c-Jun plasmids.

### Chromatin immunoprecipitation (ChIP) assay

The combination between the transcriptional factor c-Jun and ENKUE promoter was evaluated using the ChIP analysis kit (Thermo Scientific, Waltham, MA, USA). DNA fragments were obtained after chromatin was cross-linked, separated, and digested with micrococcal nuclease. The IgG or anti-c-Jun was mixed in the reaction system for immunoprecipitation. The recovered DNA fragments were eluted, purified, then detected using qPCR assays.

### Electrophoretic mobility shift assay (EMSA)

EMSA kit (BersinBio, Guangzhou, China) was used to determine the electrophoretic mobility change following the manufacturer's instructions. Nuclear contents were extracted, and the concentration was examined using a BCA analysis kit. A reaction mixture, including nuclear content and biotin-labeled probes, was used for EMSA. Cold competitors (mutant and unlabeled wild probes) (Supplementary Table 3) and c-Jun antibody were added to this reaction content for competition and super-shift assays. Finally, the signal was detected and analyzed.

### Co-immunoprecipitation (Co-IP)

Pierce Co-Immunoprecipitation kit (Thermo Scientific, Waltham, MA, USA) was used to obtain proteins from the cells. BCA kit was used to determine protein concentration following manufacturer's instructions. Protein (5 mg) was mixed with 10 µg antibodies or IgG (negative control) and incubated at 4 °C overnight. Finally, after eluting the mixed products, the recovered protein was used for Western blot analysis.

### Cycloheximide (CHX) chase assay

HCC cells transfected with ENKUR or MYH9 plasmids were incubated with or without 20 μmol/l MG132 (Sigma-Aldrich, MO, USA) for 6 hours. The cells were then mixed with 50 µg/ml CHX (Abcam, Massachusetts, USA) and collected every 10 minutes for western blots.

### Tissue specimens

Tumor and para tumorous tissue of Twenty-three (23) paraffin-embedded HCC tissue sections were obtained from surgical resections of patients admitted at the Integrated Hospital of Traditional Chinese Medicine, Southern Medical University. Two HCC tissue microarrays containing 180 paraffin-embedded HCC specimens and corresponding peritumoral tissues were obtained from Outdo corporation, Shang Hai, China. All the patients included did not undergo any treatment before the specimen was obtained (http://www.superchip.com.cn/). The local ethics committee approved the study. Thirteen of one hundred and eighty samples with more than 30% missing data were excluded.

### Immunohistochemistry (IHC)

The paraffin section was first deparaffinized and dehydrated. Antigen retrieval was then achieved using citrate buffer for 3 minutes. Endogenous peroxidase and non-specific antigens were inhibited using 3% H_2_O_2_ and goat serum. The antigens were incubated with various antibodies at 4 °C overnight. The sections were washed and incubated with an HRP-conjugated secondary antibody. DAB kit (Maixin Biotech. Co., Ltd, Fuzhou, China) was then used to visualize the sections. A score ≤ 4 indicated low expression, whereas a score > 4 indicated high expression.

### Statistical analysis

SPSS 23.0 (SPSS, Inc. Chicago, IL, USA) was for all statistical analysis. Data are expressed as mean ± SD. A student's two-tailed t-test was used to determine the statistical difference between the two groups. One-way analysis of variance was used to compare the statistical differences between multiple groups. A general linear model was used to measure tumor growth and MTT. Wilcoxon rank-sum test was used to analyze skewed data. Log-rank tests were conducted using Kaplan-Meier survival curves to illustrate the significant association between gene expression and overall survival of HCC patients. Univariate and multivariate survival analyses were conducted through Cox proportional hazards regression model. P<0.05 was considered statistically significant.

## Results

### Decreased ENKUR levels are correlated with unfavorable outcome in HCC patients

**Figure 1:**
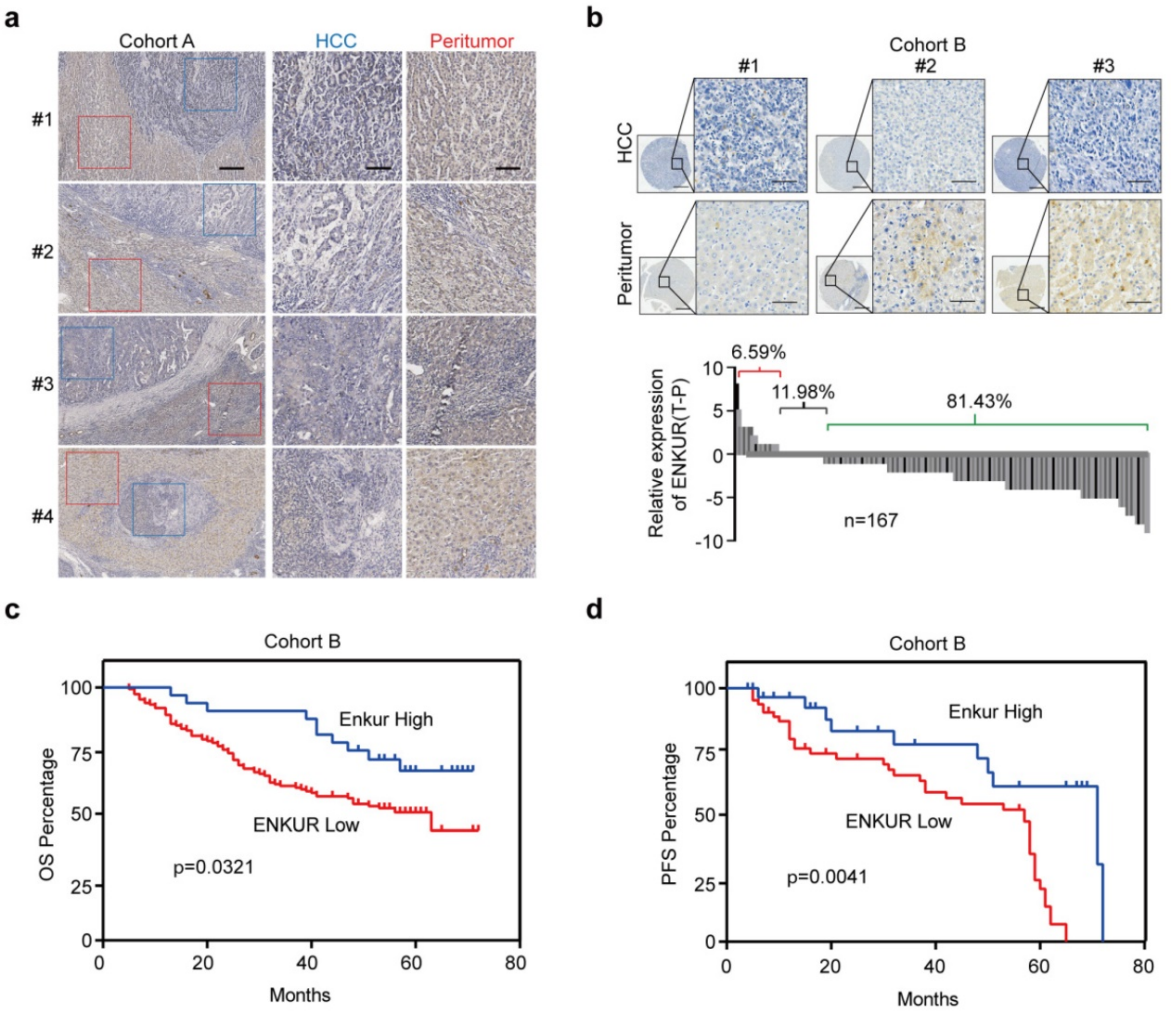
** The relationship between ENKUR expression and HCC progression and patient prognosis. (a)** IHC staining intensities of ENKUR in liver cancer and adjacent tissues (scale bar: 250 µm). **(b)** IHC staining intensities of ENKUR assessed using two tissue arrays containing 167 of HCC patients (scale bar: 250 µm). **(c)** Overall survival (OS) and disease free survival (PFS, **d**) of HCC patients as conducted by Kaplan-Meier survival analysis based on ENKUR expression.

In this study, UALCAN (http://ualcan.path.uab.edu/index.html), a webserver for analyzing TCGA data 27,28 was used to analyze ENKUR level in HCC and control liver tissues to explore its clinical significance. ENKUR mRNA level was significantly downregulated in HCC tissues (p<0.001, Figure. S1a). Immunohistochemistry (IHC) was performed using 23 HCC tumors containing adjacent non-cancerous tissues obtained from our hospital. ENKUR protein expression level was significantly down-regulated in HCC tissues compared with normal cells (Figure 1a). Two tissue arrays containing 167 of HCC patients were used to explore ENKUR expression further. Similarly, 81.43% of the patients had lower ENKUR expression levels in HCCs (Figure 1b). Notably, Kaplan-Meier analysis showed that reduced ENKUR levels were correlated with worse overall survival (OS) and disease-free survival (PFS) in HCC patients (Figure 1c, d). These findings show that ENKUR can be used as a biomarker in HCC patients.

### ENKUR inhibits cell proliferation, metastasis, and sorafenib resistance in HCC patients

**Figure 2:**
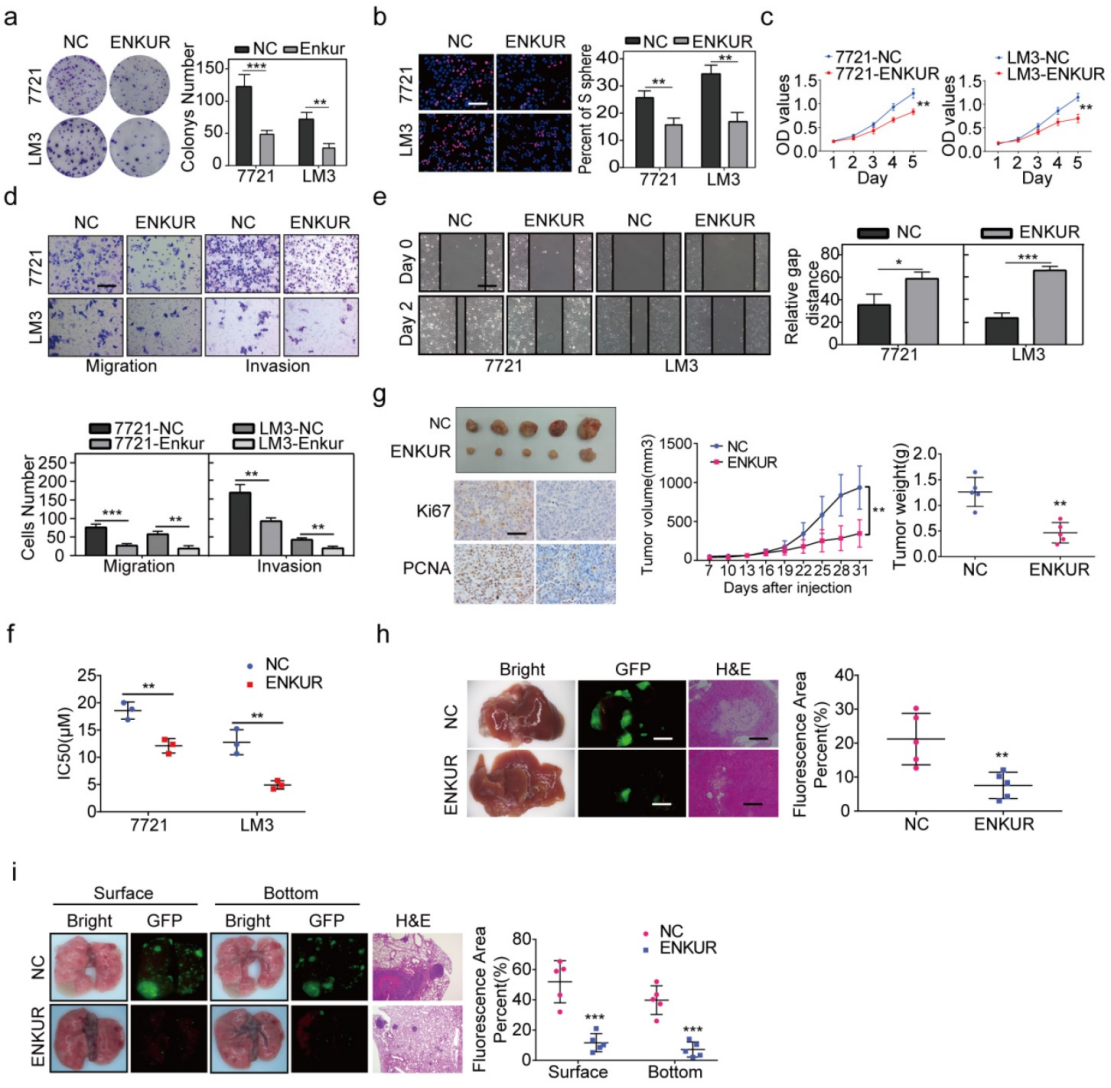
** ENKUR inhibits HCC proliferation, metastasis, and sorafenib resistance. (a)** Colony formation assays; **(b, c)** Edu (scale bar: 100 µm) and MTT analyses; **(d, e)** invasion, migration (scale bar: 10 µm) and wound healing assays (scale bar: 10 µm) of ENKUR-overexpressing 7721 and LM3 cells and the corresponding control cells. (f) Dose-dependent and time-dependent growth curves of 7721 and LM3 cells treated with sorafenib. **(g)** A mouse subcutaneous tumor model used to explore the effect of ENKUR on proliferation (n=5, general linear model). Tumor paraffin sections were stained with immunohistochemistry (scale bars: 50 µm) to determine Ki67 and PCNA expression levels (n=5). **(h, i)** Mouse *in situ* tumor model used to explore the effect of ENKUR on proliferation and metastasis (n=5). Student's t-test was used to compare the treatment groups vs. the control group (n=3 independent experiments). Data were presented as mean ± SD. H&E scale bar: 50 µm.

RT-qPCR and western blot assay were used to evaluate ENKUR expression in HCC and non-cancerous liver cell lines. The mRNA and protein expression levels of ENKUR were lower in HCC cell lines than in the non-cancerous cell lines (Figure S2a). Lentivirus stably overexpressed ENKUR in low ENKUR-expressing 7721 and LM3 cells (Figure S2b). Overexpression efficiency of lentivirus was also confirmed at the protein level (Figure S2b). Various *in vitro* assays were used to evaluate the effect of ENKUR overexpression on cell functions, including proliferation, migration, invasion, and sorafenib resistance, to explore whether ENKUR is functionally involved in HCC cells. Colony formation assays showed that ENKUR-overexpression suppressed HCC cell proliferation (Figure 2a). Edu and MTT assays also confirmed the inhibitory effect of ENKUR overexpression on HCC cell proliferation (Figure 2b, c). Moreover, transwell and boyden assays indicated that the ENKUR overexpression in 7721 and LM3 cells suppressed the cell migration and invasion (Figure 2d). Wound healing migration assay showed that ENKUR overexpression delayed wound healing in HCC cells (Figure 2e). Moreover, ENKUR overexpression significantly reduced half-maximal inhibitory concentration (IC50) of sorafenib (Figure 2f). In contrast, ENKUR silencing using siRNA (Figure S2c) promoted HCC proliferation (Figure S2d), metastasis (Figure S2e), and sorafenib resistance (Figure S2f). These findings show that ENKUR inhibits HCC proliferation, metastasis, and sorafenib resistance *in vitro*.

Furthermore, *in vivo* experiments were used to evaluate the anti-tumor efficacy of ENKUR. The subcutaneous xenograft mouse model showed that tumors derived from ENKUR-overexpressing HCC cells were significantly smaller and lighter than those from the negative control group (Figure 2g). IHC assays showed that the expression levels of PCNA and Ki67 in tumors were significantly lower in the ENKUR-overexpressing group than in the control group (Figure 2g). Similarly, orthotopic transplantation tumor model showed that ENKUR-overexpressing HCC cells inhibited tumor growth in mice (Figure 2h). These findings show that ENKUR suppresses HCC proliferation *in vivo*. Moreover, a pulmonary metastasis model was established to assess the metastasis function of ENKUR *in vivo*. Fluorescence and HE assays showed that ENKUR-overexpressing HCC cells reduced metastatic nodules in mice compared with the control group (Figure 2i).

### ENKUR interacts with β-catenin to suppress its nuclear accumulation

**Figure 3:**
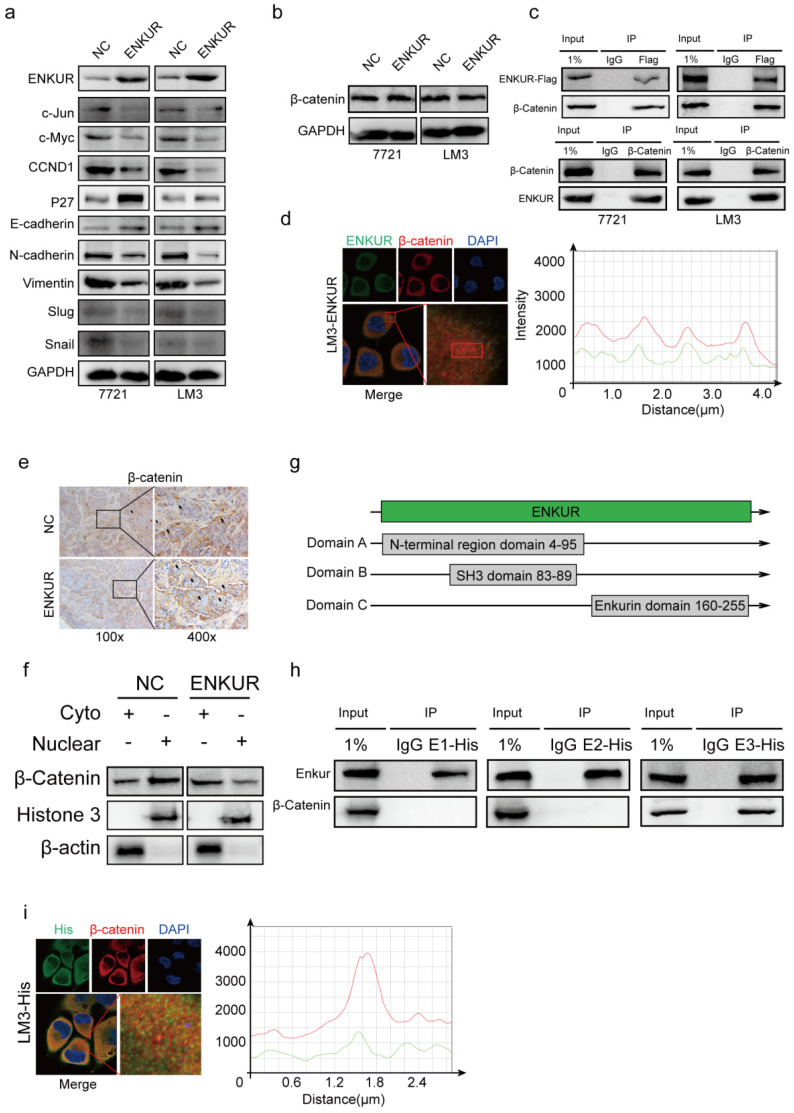
** ENKUR interacts with β-catenin to suppress the accumulation of nuclear β-catenin. (a, b)** Western blot analysis showing the expression levels of proliferation and metastasis-associated proteins in HCC cells; **(c)** Co-immunoprecipitation (Co-IP) assay showing the interaction between ENKUR and -catenin in LM3 and 7721 cells overexpressing ENKUR; **(d)** Immunofluorescence (IF) assays showing the co-localization of ENKUR and β-catenin protein in the cytoplasm of ENKUR-overexpressing HCC cells. The red or green curves mean β-catenin or ENKUR respectively; **(e)** Immunochemistry (IHC) showing the translocation of β-catenin in tumor paraffin sections derived from subcutaneous xenograft mouse model; **(f)** The expression of β-catenin in the cytoplasm and nucleus of HCC cell determined with or without ENKUR overexpression. **(g)** Functional domains of ENKUR; Co-IP **(h)** and IF **(i)** assays showing the interaction between Enkurin domain of ENKUR and β-catenin. The red or green curves mean β-catenin or Enkurin domain.

Western blot assays showed that ENKUR overexpression down-regulated c-Jun, c-Myc, CCND1, N-cadherin, Vimentin, Snail, Slug, while it upregulated P27 and E-cadherin (Figure 3a). However, ENKUR overexpression did not significantly affect β-catenin expression (Figure 3b). Co-immunoprecipitation (Co-IP) showed that ENKUR interacted with β-catenin in HCC cells (Figure 3c). Immunofluorescence (IF) was used to assess the protein co-localization between ENKUR and β-catenin in the cytoplasm of HCC cells (Figure 3d). Immunochemistry (IHC) assay showed that ENKUR overexpression inhibited translocation of β-catenin from the cytoplasm to the nucleus in paraffinized tumor sections from the subcutaneous xenograft mouse model (Figure 3e). Moreover, western blot showed that ENKUR overexpression inhibited the nuclear entrance of β-catenin in LM3 (Figure 3f). Plasmids of the three functional domains of ENKUR were constructed using domain information obtained from UniProt (https://www.uniprot.org/uniprot/Q8TC29#family_and_domains) to further explore the interaction between ENKUR and β-catenin (Figure 3g). Co-IP and IF assays showed that the Enkurin domain interacted with β-catenin (Figure 3h, i). These findings indicate that ENKUR interacts with β-catenin to suppress the accumulation of nuclear β-catenin, cell cycle, and EMT signaling in HCC cells.

### Enkurin domain mediates binding of ENKUR to MYH9

**Figure 4:**
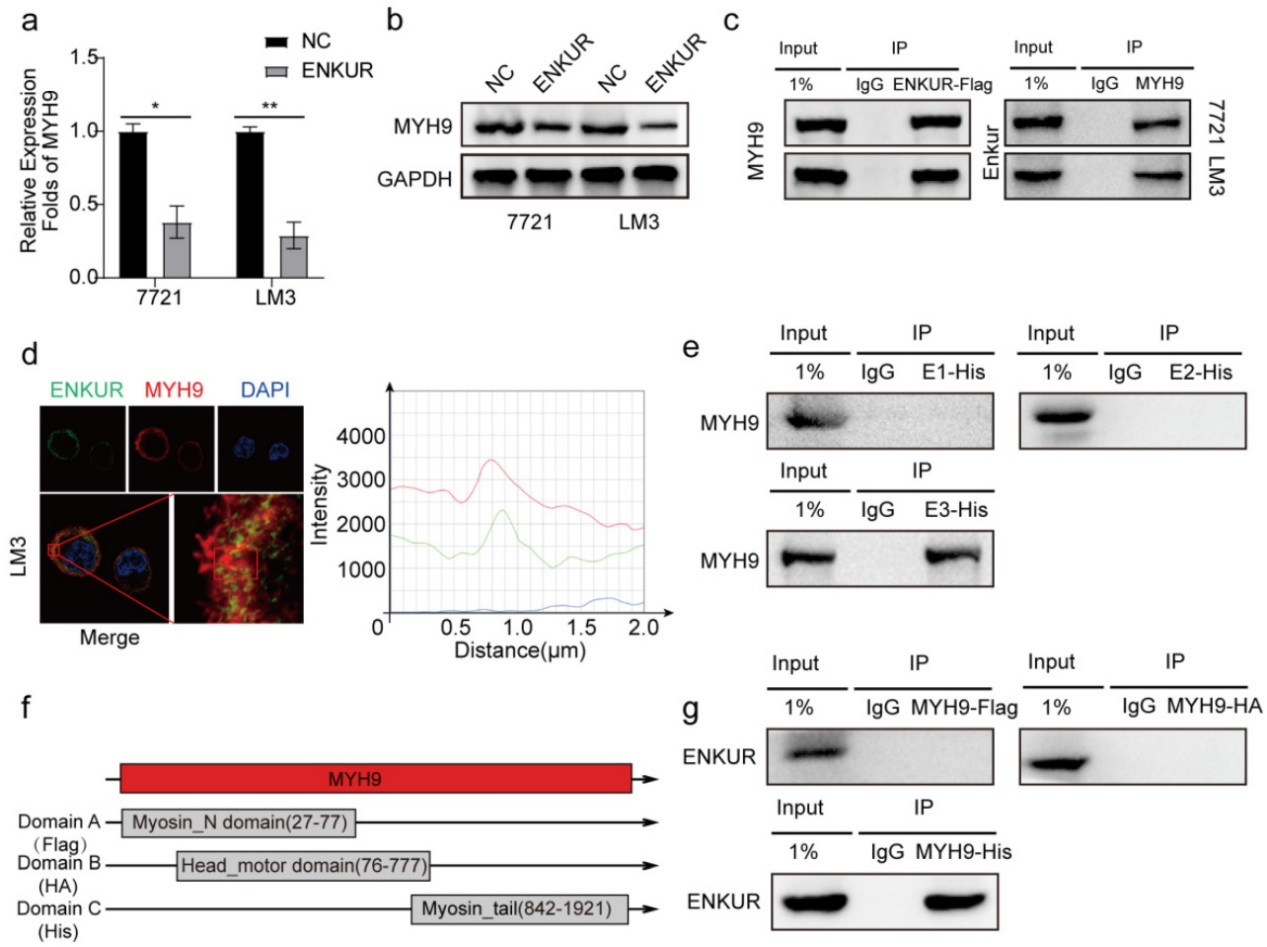
** Enkurin domain mediates binding of ENKUR to MYH9. (a, b)** The mRNA and protein levels of MYH9 in HCC cells with or without ENKUR. **(c)** Co-IP analysis showing the interaction between ENKUR and MYH9 in ENKUR-overexpressing HCC cells. **(d)** Co-localization of ENKUR and MYH9 in ENKUR-overexpressing HCC cells; The red or green curves mean MYH9 and ENKUR respectively. **(e)** Co-IP analysis showing the interaction between ENKUR domains and MYH9 in HCC cells transfected with plasmids containing different ENKUR domains. **(f)** A schematic diagram of MYH9 domains. **(g)** Co-IP assays showing the interaction between domains and ENKUR in HCC cells transfected with plasmids containing different MYH9 domains.

Herein, qPCR assay and western blot showed that ENKUR decreased the mRNA and protein expressions of MYH9 (Figure 4a, b). After Co-IP and mass spectrometry analysis showed that MYH9 was a potential ENKUR-interacting protein with relatively high match scores in ENKUR-overexpressing A549 cells (The data is only available on inquiry because we have another unpublished paper). Then Co-IP assays indicated that ENKUR interacts with MYH9 (Figure 4c) and that ENKUR and MYH9 were colocalized in the cytoplasm via immunofluorescence assay (Figure 4d). Co-IP assay was performed in LM3 cells with different domain plasmids to determine whether ENKUR interacts with MYH9 through a specific domain. The results showed that the interaction between ENKUR and MYH9 occurred through the Enkurin domain (Figure 4e). The myosin tail domain, one of the three domains of MYH9, also interacted with ENKUR in HCC cells (Figure 4f, g). Then we found that the inhibition of ENKUR was reversed after MYH9 plasmids were transfected into 7721 and LM3 via boyden assay, wound healing assay and EDU assay (Figure S3a-c). These findings show that ENKUR and MYH9 interact through the Enkurin domain and the myosin tail domain.

### ENKUR attenuates tumor cycle and EMT signaling by suppressing MYH9/USP7-mediated c-Myc deubiquitylation and stability

**Figure 5:**
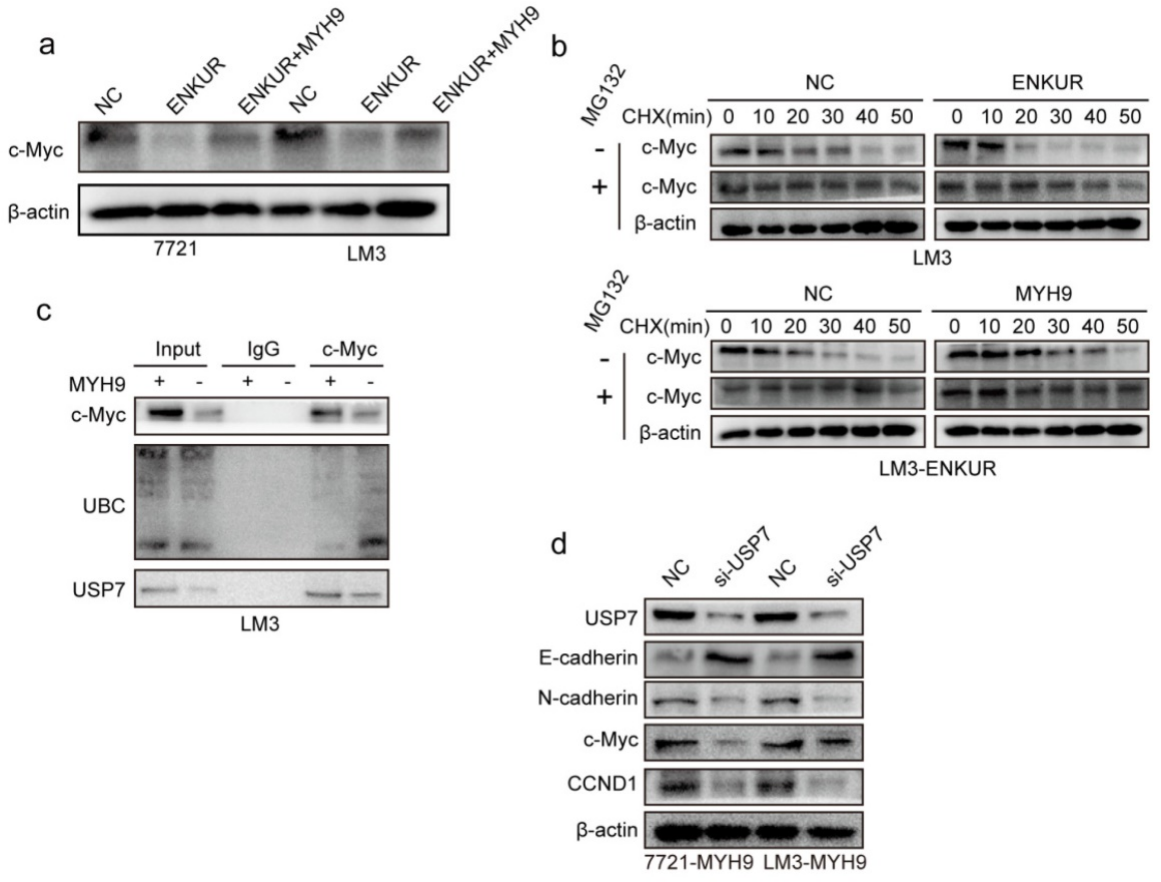
** ENKUR attenuates tumor cycle and EMT signaling by suppressing MYH9/USP7-mediated c-Myc deubiquitination and stability. (a)** Protein expression level of c-Myc; **(b)** Western blot showing the effect of ENKUR and MYH9 on c-Myc stability in LM3 cells incubated with cycloheximide with or without MG132 at different time points. **(c)** Interactions among MYH9, USP7 and Ubiquitin in HCC cells. **(d)** Protein changes in MYH9-overexpressing LM3 cells transfected with si-USP7.

C-Myc is a well-characterized oncoprotein involved in HCC 29,30. Herein, western blot assays showed that ENKUR overexpression inhibited protein expression of c-Myc. However, the protein expression of c-Myc was restored when MYH9 plasmid was introduced into ENKUR-overexpressing cells (Figure 5a). A mass spectrometric analysis published in Biogrid (https://thebiogrid.org) showed that MYH9 interacts with USP7, a deubiquitination enzyme for deubiquitylation, thus stabilizing c-Myc 31. Therefore, these results indicate that ENKUR regulates c-Myc degradation by suppressing the MYH9-mediated recruitment of USP7. Herein, cycloheximide was introduced into ENKUR-overexpressing LM3 and control cells, and the results showed that ENKUR decreased stabilization of c-Myc. Moreover, MYH9 upregulation extended the half-life of c-Myc in ENKUR-overexpressing LM3 cells (Figure 5b). Co-IP assays showed that MYH9 recruited USP7 to form a deubiquitin ligase complex, thus deubiquitinating and stabilizing c-Myc (Figure 5c). Moreover, USP7 suppression downregulated c-Myc, thus inhibiting the tumor cycle and EMT signaling in HCC cells transfected with MYH9 plasmids (Figure 5d).

### C-Jun inhibits ENKUR by binding to its promoter region

**Figure 6:**
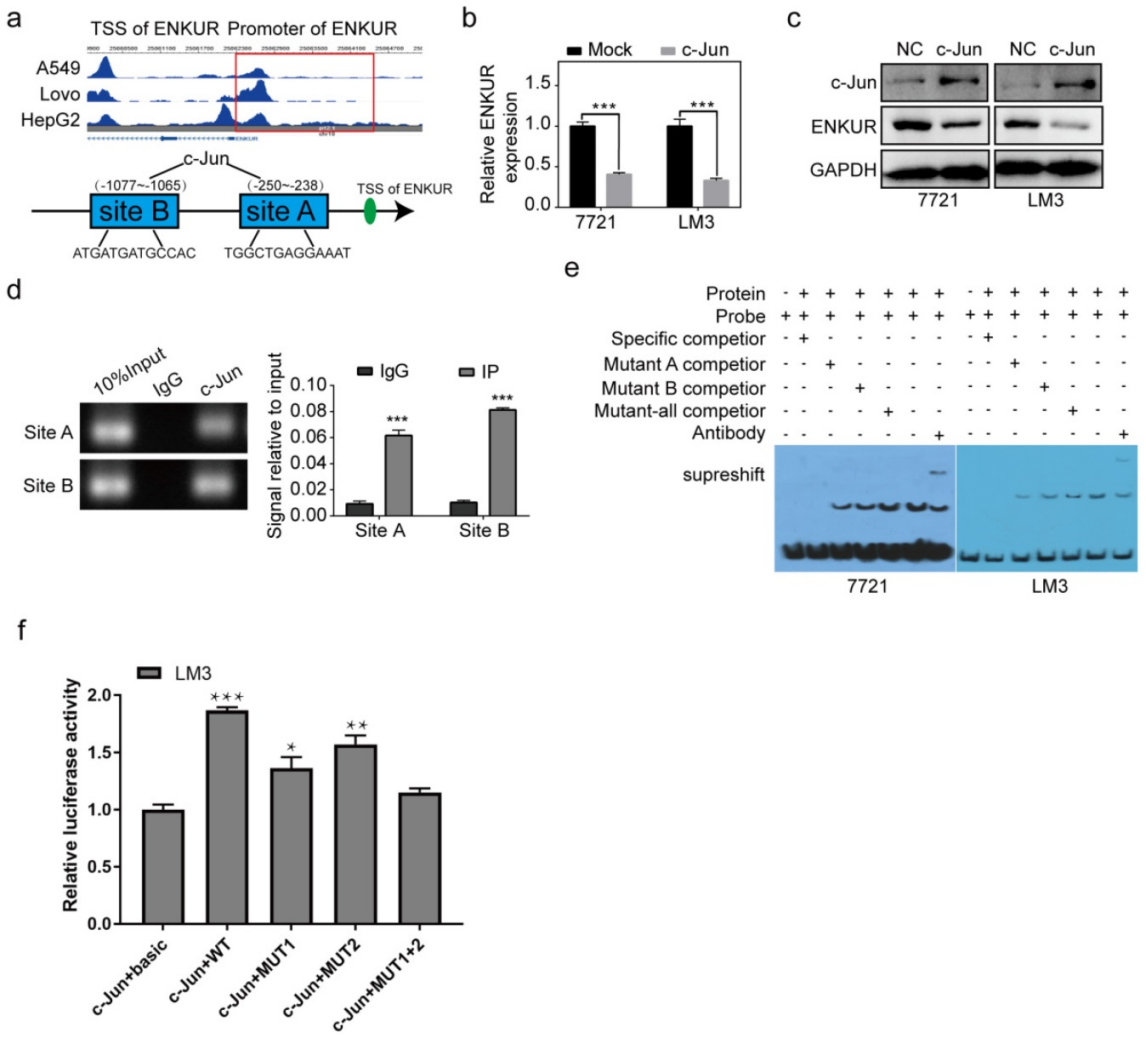
** c-Jun inhibits ENKUR by binding to its promoter region. (a)** The ChIP-seq binding peak in the Cistome database; Bioinformatics website predicted the c-Jun binding site in the ENKUR transcriptional regulatory region; **(b,c)** mRNA and protein expression levels of ENKUR in HCC cells transfected with c-Jun plasmids and control cells; **(d)** ChIP assays showing the binding of c-Jun to ENKUR promoter in HCC cells. **(e)** Gel electrophoresis showing c-Jun binding to ENKUR promoter in HCC cells after ChIP and electrophoretic mobility shift assays (EMSA). **(f)** Dual-luciferase assay showing the binding of c-Jun to ENKUR promoter in HCC cell.

Herein, two c-Jun binding sites were identified inside the transcriptional regulatory region of ENKUR using the Cristome Data Browser and JASPAR (Figure 6a). C-Jun overexpression significantly decreased ENKUR expression (Figure 6b, c). ChIP assay showed that endogenous c-Jun could bind to ENKUR promoter in HCC cells (Figure 6d). EMSA data and dual luciferase assay further showed that c-Jun specifically binds to the ENKUR promoter (Figure 6e, f).

### CB inhibits tumor proliferation, metastasis, and sorafenib resistance in HCC

Previous findings revealed that CB inhibits tumor proliferation, metastasis, and chemotherapy resistance through PI3K/AKT axis in NPC 22. As a result, the role of CB as an anti-tumor factor in HCC was explored. The chemical structure formula of CB is shown in Figure S4a. MTT assays showed that the IC50s of CB in SMMC-7721 and HCC-LM3 were 480.5 nM and 655.4 nM, respectively (Figure S4b). The IC50 concentrations were selected for follow-up experiments. Cell culture showed that CB suppressed HCC cell proliferation concentration-dependent (Figure S4c). Moreover, boyden and wound healing assays showed CB suppressed HCC invasion and migration (Figure S4d, e). A low concentration of CB decreased the IC50 of sorafenib in HCC cells (Figure S4f). Western blot analysis showed that CB inhibited p-PI3K, p-AKT, c-Jun, N-cadherin, and Vimentin expression while inducing E-cadherin expression (Figure S4g). These findings indicate that CB suppresses tumor activities by inhibiting PI3K/AKT/c-Jun axis *in vitro*.

**Figure 7:**
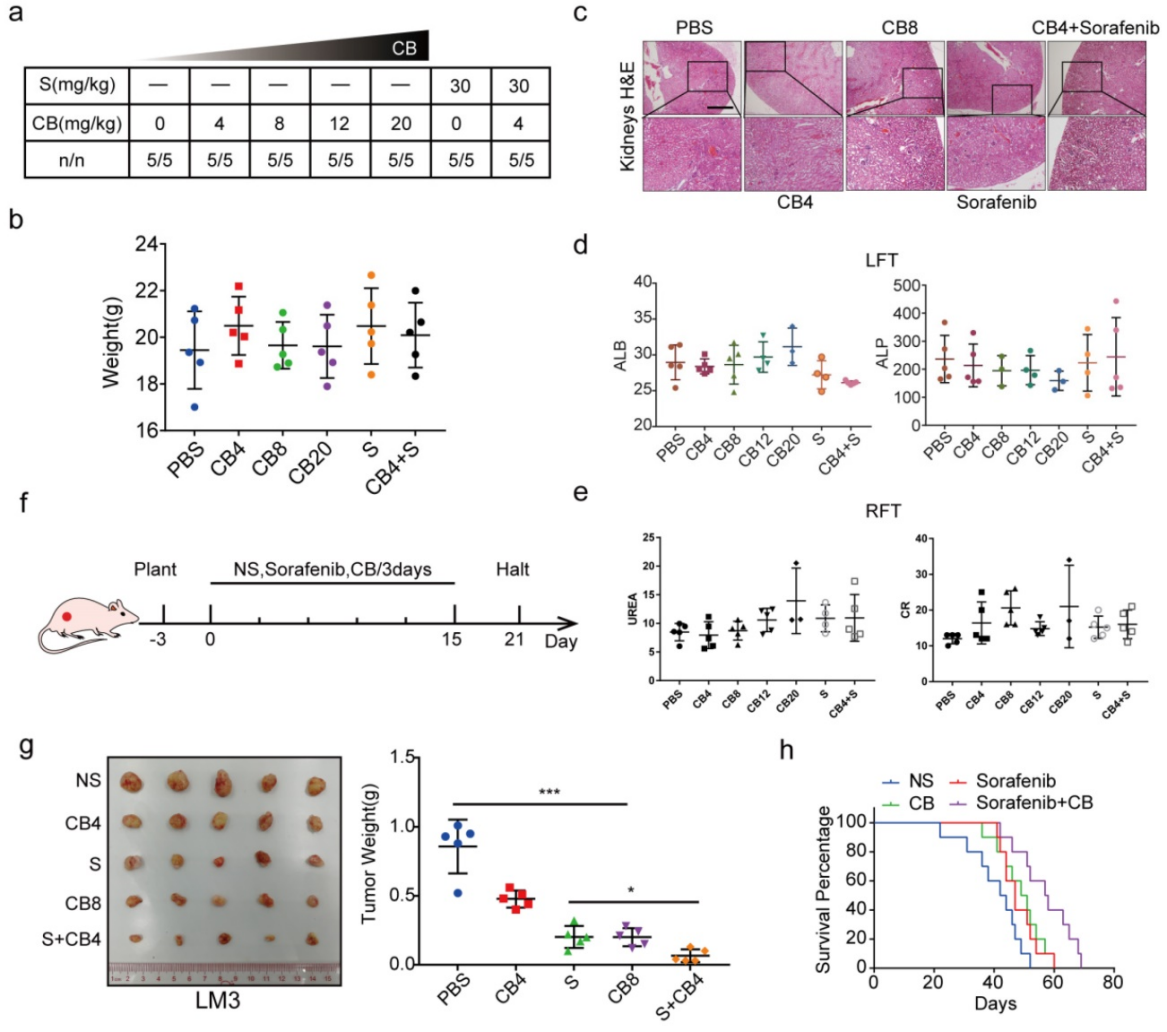
** CB inhibits tumor proliferation, metastasis, and sorafenib resistance in HCC. (a, b)** Toxicity and effectiveness assays of CB; **(c)** Liver function tests (LFT) and Renal function tests (RFT) showing drug-induced liver and renal injury of CB, respectively; **(d, e)** Levels of liver function biochemical indexes; **(f)** Flow chart of animal experiments; **(g)** Tumors of the five treatment groups; **(h)** Survival time of nude mice treated with a combination of sorafenib and CB dose.

*In vivo* experiments were also used to explore the toxicity and effectiveness of CB. The seven groups of nude mice were intraperitoneally administered with five concentrations of CB (0, 4, 8, 12, 20 mg/kg) and sorafenib (30 mg/kg) for three weeks. All mice survived and had no significant body weight change (Figure 7a, b). The CB-induced liver and renal injuries were assessed using liver function test (LFT) and renal function test (RFT), respectively. Hematoxylin and eosin staining (H&E) showed that kidneys from the CB-treated mice had no pathological changes (Figure 7c). Moreover, serum was collected from mice in the seven groups for liver and kidney function tests. The levels of liver function biochemical indexes, including ALP and ALB, slightly changed in all treatment groups (Figure 7d). Similarly, the levels of creatinine (CR) and urea, the key renal function indexes, were not significantly altered in all treatment groups (Figure 7e). Moreover, a subcutaneous tumor mouse model was developed using five treatment groups (NS, 0, 4, 8 mg/kg of CB and 30 mg/kg of sorafenib) (Figure 7f). Therapeutic effect of the 8 mg/kg CB group and the sorafenib group was not significantly different. Interestingly, the 4 mg/kg CB combined with the sorafenib group had the smallest tumors (Figure 7g). Notably, nude mice in the sorafenib combined with CB group had the longest survival time (Figure 7h). These results show that a low CB dose combined with sorafenib is safe and effective for HCC treatment.

### CB enhances ENKUR-mediated anti-tumor activities by modulating PI3K/AKT/c-Jun axis

**Figure 8:**
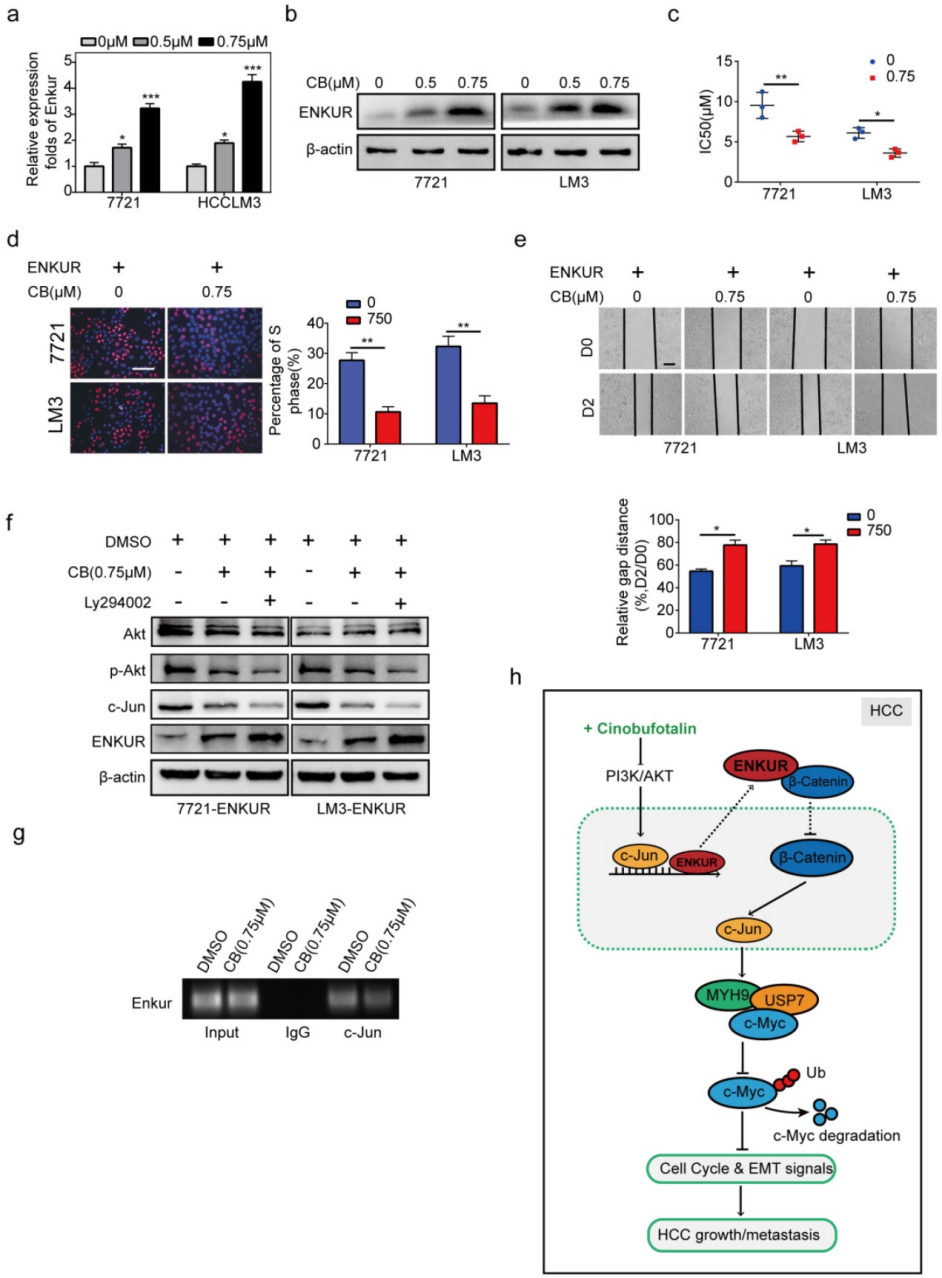
** CB enhances ENKUR-mediated anti-tumor activities by regulating PI3K/AKT/c-Jun axis.** ENKUR mRNA **(a)** and protein **(b)** levels in HCC cells treated with CB. dose-dependent growth curves and time-dependent growth curves **(c)**, Edu assay (**d,** scale bar: 10 µm), Transwell assay (e, scale bar: 10 µm), of ENKUR-overexpressing 7721 and LM3 cells treated with CB; **(f)** Expression levels of vimentin, N-cadherin, and E-cadherin in ENKUR-overexpressed HCC cells after CB treatment; **(g)** ChIP assays showing the binding of c-Jun to ENKUR promoter in HCC cells treated with CB; **(h)** Working model indicating how CB suppresses HCC malignant activities by antagonizing ENKUR-mediated β-catenin/c-Jun/MYH9/USP7 pathway, thus increasing c-Myc ubiquitin degradation.

The effect of CB on mRNA and protein levels of ENKUR in HCC cells was determined to explore the effect of CB on ENKUR-induced inhibition of tumor activities (Figure 8a, b). CB induced ENKUR to inhibit HCC proliferation, metastasis, and sorafenib resistance (Figure 8c-e). Western blot assay demonstrated that the CB-treated ENKUR-overexpressed HCC cells with CB suppressed the levels of p-AKT and c-Jun. The same expression levels were detected after the addition of the PI3K inhibitor Ly294002 to CB-treated ENKUR-expressed HCC cells (Figure 8f). ChIP assays showed that CB-treated HCC cells significantly enhanced the binding activity of c-Jun to ENKUR promoter (Figure 8g). These findings show that CB enhances ENKUR activity by regulating PI3K/AKT/c-Jun. As a result, the interaction between ENKUR and β-catenin is increased to antagonize β-catenin-stimulated tumor growth and EMT signals.

## Discussion

Previous studies had showed that ENKUR is a potential tumor suppressor participating the pathogenesis of lung adenocarcinoma and colorectal cancer cells 12,13. However, the action and molecular mechanisms of ENKUR in HCC are unknown. Here, ENKUR was significantly downregulated in HCC compared with the para-cancer tissues, indicating that ENKUR expression is an independent predictive factor for the outcome of HCC patients. Furthermore, ENKUR repressed HCC growth, migration, invasion, and metastasis *in vitro* and *in vivo*. ENKUR overexpression enhanced the sensitivity of HCC cells to sorafenib therapy *in vitro* and prolonged the survival time of tumor-bearing mice treated with sorafenib. These results suggest that ENKUR can inhibit HCC.WNT/β-catenin signaling regulates tumor EMT, growth, and sorafenib resistance in HCC 32,33. Wnt/β-catenin signaling is silenced in the normal liver of adults via a balanced modulation of Wnt ligands and β-catenin destruction complex 34. Wnt ligand induces β-catenin release from GSK-3β/APC/AXIN1 complex to HCC nucleus, thus activating TCF/LEF family transcription factors and their downstream signals 35. Herein, ENKUR did not regulate the key factor β-catenin expression of Wnt/β-catenin signaling at mRNA and protein levels. Further experiments showed that the ENKUR and β-catenin interacted in the ENKUR-overexpressed HCC cells. Moreover, ENKUR/β-catenin complex formation inhibited β-catenin translocation to the nucleus, thus suppressing β-catenin-modulated HCC progression. β-catenin also interacted with the IQ motif domain of ENKUR. These findings indicate that ENKUR induces anti-tumor effect by inhibiting β-catenin-induced tumor activities.

In order to deeply investigate the molecular basis of ENKUR involved in the pathogenesis of HCC, we used CoIP combined with mass spectrometry assays to find that MYH9 is a candidate interactive protein of ENKUR in ENKUR-overexpressing A549 cells (data not published). Further, Co-IP and immunofluorescence assays showed that ENKUR interacted with MYH9 in the cytoplasm. The domain is an independent folding unit in the tertiary structure of a protein with a unique spatial conformation and different functions. Herein, ENKUR induced an anti-metastasis role by interacting with MYH9 through Enkurin domain. Notably, MYH9 also interacted with ENKUR through the myosin tail domain. β-catenin/c-Jun-dependent transcriptional regulation of MYH9 has been reported in HCC 23. Herein, ENKUR-overexpressing HCC cells downregulated mRNA and protein expression levels of MYH9. Subsequent mechanistic analysis revealed that ENKUR interacted with β-catenin, attenuating its nuclear translocation, thus inhibiting c-Jun-dependent positive transcriptional regulation of MYH9 in HCC cells. These findings show that ENKUR interacts with MYH9 and reduce its expression by binding to β-catenin, thus inhibiting its translocation to the nucleus and decreasing c-Jun transcription.

C-Myc is a key tumor suppressor that inhibits tumor cycle and EMT signaling in many cancers. Ubiquitination is a posttranslational protein modification method. Ubiquitin-mediated c-Myc stability promotes cancer development 31,36,37. Herein, ENKUR suppressed c-Myc stability. However, MYH9 reversed the effect of ENKUR. A previous study reported that deubiquitination enzyme USP7 binds to c-Myc protein, thus deubiquitinating and stabilizing c-Myc protein. Herein, MYH9 recruited USP7 and c-Myc, thus suppressing ubiquitin-mediated degradation of c-Myc. These findings show that ENKUR binds to MYH9 and decreases its protein expression, thus reducing recruitment of the deubiquitination enzyme USP7 and inducing the ubiquitination level of c-Myc protein. As a result, c-Myc protein is degraded, resulting in the downregulation of cell cycle and EMT signalings. Thesedata show that ENKUR suppresses HCC cell proliferation and metastasis by inactivating β-catenin/c-JUN/MYH9/USP7/c-Myc-stimulated cell cycle and EMT signalings.

Previous studies have reported that aberrant transcriptional regulation of c-Jun can promote the pathogenesis of various diseases 38-40. Moreover, c-Jun is essential in HCC growth, metastasis, and sorafenib resistance 41,42. Interestingly, bioinformatics analysispredicted that c-Jun is a transcriptional factor of ENKUR. Herein, c-Jun transcriptionally downregulated ENKUR in HCC. This study provides a novel mechanism for ENKUR inactivation and demonstrates the role of c-Jun/ENKUR axis in HCC.

Drugs can be obtained from active compounds of natural flora and fauna 43,44. For instance, artemisinin is a sesquiterpene lactone derived from the herb Qinghao and is widely used as an antimalarial drug 45,46. Paclitaxelis also extracted from the yew plant and is used to treat ovarian, breast, and lung cancer 47-49. Cinobufotalin (CB) is derived from toad venom. Previous work revealed that CB is a promising anti-tumor agent in NPC. Herein, mice treated with different doses of CB and sorafenib had no significant body weight changes. HE staining showed no acute liver toxicity 50 and acute kidney injury (AKI) in mice 51. Moreover, the serum levels of LFT and RFT indexes slightly changed in the CB treatment groups. However, the changes were significantly lower than the diagnostic criteria for drug-induced liver injury (DILI) 52 and drug-induced kidney injury 53,54, indicating that CB is safe. CB had significantly higher cytotoxicity in HCC cells than sorafenib. Functional experiments showed that CB inhibited proliferation, invasion, and metastasis of HCC. Moreover, CB reduced chemoresistance of sorafenib in HCC cells. The subcutaneous tumor model showed that tumor sizes were smaller in the low-dose CB combined with the sorafenib group than in the other groups. CB significantly increased the overall survival time of nude mice than the nude mice treated with the same dosage of sorafenib. Besides, the same dose of CB elevated the survival time of nude mice compared with sorafenib or CB treatment. This is the first study to show the anti-cancer effect of CB in HCC.

A previous study reported that marinobufagenin, a CB analog, significantly increases ENKUR mRNA expression level in human brain microvascular endothelial cells 55. Herein, we also observed that CB also significantly increases ENKUR mRNA and protein expression in HCC. In previous studies, we had confirmed that CB suppresses the expression of PI3K/AKT/c-Jun 22,24, a classically oncogenic signaling pathway involved in tumor growth, metastasis, and drug resistance 56-59. Interestingly, we had found that c-Jun can bind to ENKUR promoter and negatively regulate its expression in HCC. Therefore, we speculated that CB induces ENKUR expression by inhibiting PI3K/Akt/c-Jun signal. Subsequently, we confirmed that CB induced ENKUR levels through downregulating PI3K/AKT/c-Jun axis, which thus increased ENKUR/β-catenin complex formation and finally modulated β-catenin-related tumor growth and EMT signals.

In summary, ENKUR induces an anti-tumor effect in HCC proliferation and metastasis by binding to β-catenin and suppressing nucleocytoplasmic transport of β-catenin. As a result, it antagonizes β-catenin/c-Jun/MYH9/USP7 pathway to increase the ubiquitin degradation of c-Myc and thus decrease cell cycle and EMT signals. Furthermore, CB is a potential anti-cancer agent reducing ENKUR-suppressed tumor activities by suppressing PI3K/AKT/c-Jun axis in HCC. Our data demonstrated that ENKUR induced by CB suppresses HCC malignant activities via antagonizing β-catenin/c-Jun/MYH9/USP7/c-Myc-stimulated cell cycle and EMT pathways, which indicated the significance of ENKUR as a tumor suppressor in HCC. In addition, ENKUR could also be used as a target of CB for HCC treatment.

## Supplementary Material

Supplementary figures and tables.

## Abbreviations

ENKUR: Enkurin
NS: Normal Saline
PBS: Phosphate buffer saline
ALT: Alanine aminotransferase
AST: Aspartate aminotransferase
HCC: Hepatocellular carcinoma
CB: Cinobufotalin
ChIP: Chromatin immunoprecipitation
EMSA: Electrophoretic mobility shift assay
CHX: Cycloheximide
IHC: Immunohistochemistry
OS: Overall survival
PFS: Disease-free survival
LFT: Liver function tests
RFT: Renal function tests
ALP: Alkaline phosphatase
ALB: Albumin
CR: creatinine
