# Supplementary material for: ENKUR expression induced by chemically synthesized cinobufotalin suppresses malignant activities of hepatocellular carcinoma by modulating β-catenin/c-Jun/MYH9/USP7/c-Myc axis
Source: Int J Biol Sci. 2022 Mar 21;18(6):2553–67. doi: 10.7150/ijbs.67476 (PMC8990461; doi:10.7150/ijbs.67476)
Supplement: Supplementary file 1 — Supplementary figures and tables. [file ijbsv18p2553s1.pdf]

Supplementary Materials

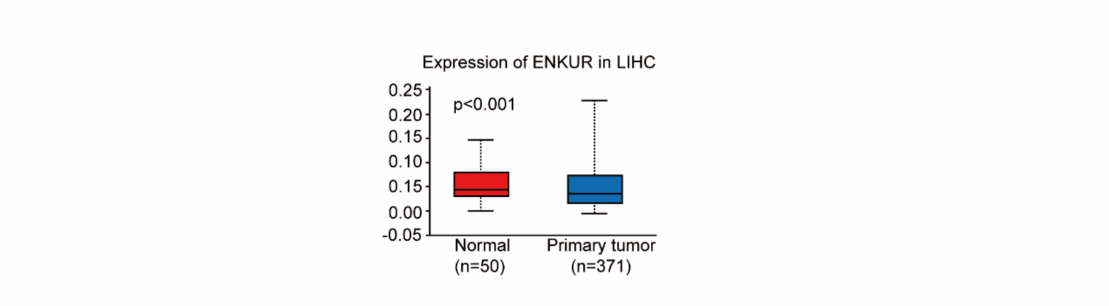

Figure S1. ENKUR expression levels of HCC patients in TCGA dataset.

Supplementary figure 2

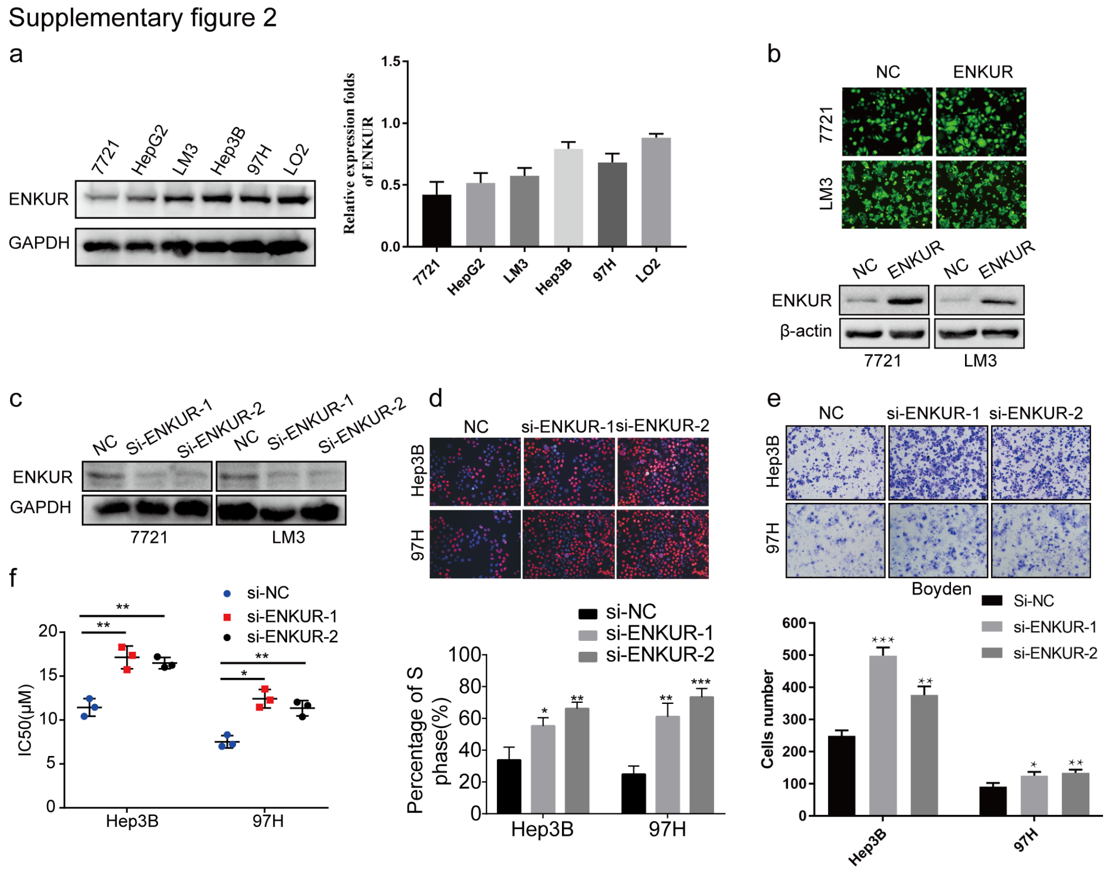

Figure S2. Suppression of ENKUR induces HCC proliferation, metastasis, and sorafenib resistance in HCC cells.

(a) mRNA and protein expression levels of ENKUR in HCC cell lines; (b) Stably overexpressed ENKUR in low ENKUR-expressing 7721 and LM3 cells via lentivirus;

(c) Protein of ENKUR in HCC cells with Si-RNA transfected; Edu assays (d, scale bar: 10 μm), Transwell assays (e, scale bar: 10 μm), and dose-dependent growth curves and time-dependent growth curves (f) of 7721 and LM3 cells treated with sorafenib.

Supplementary figure 3

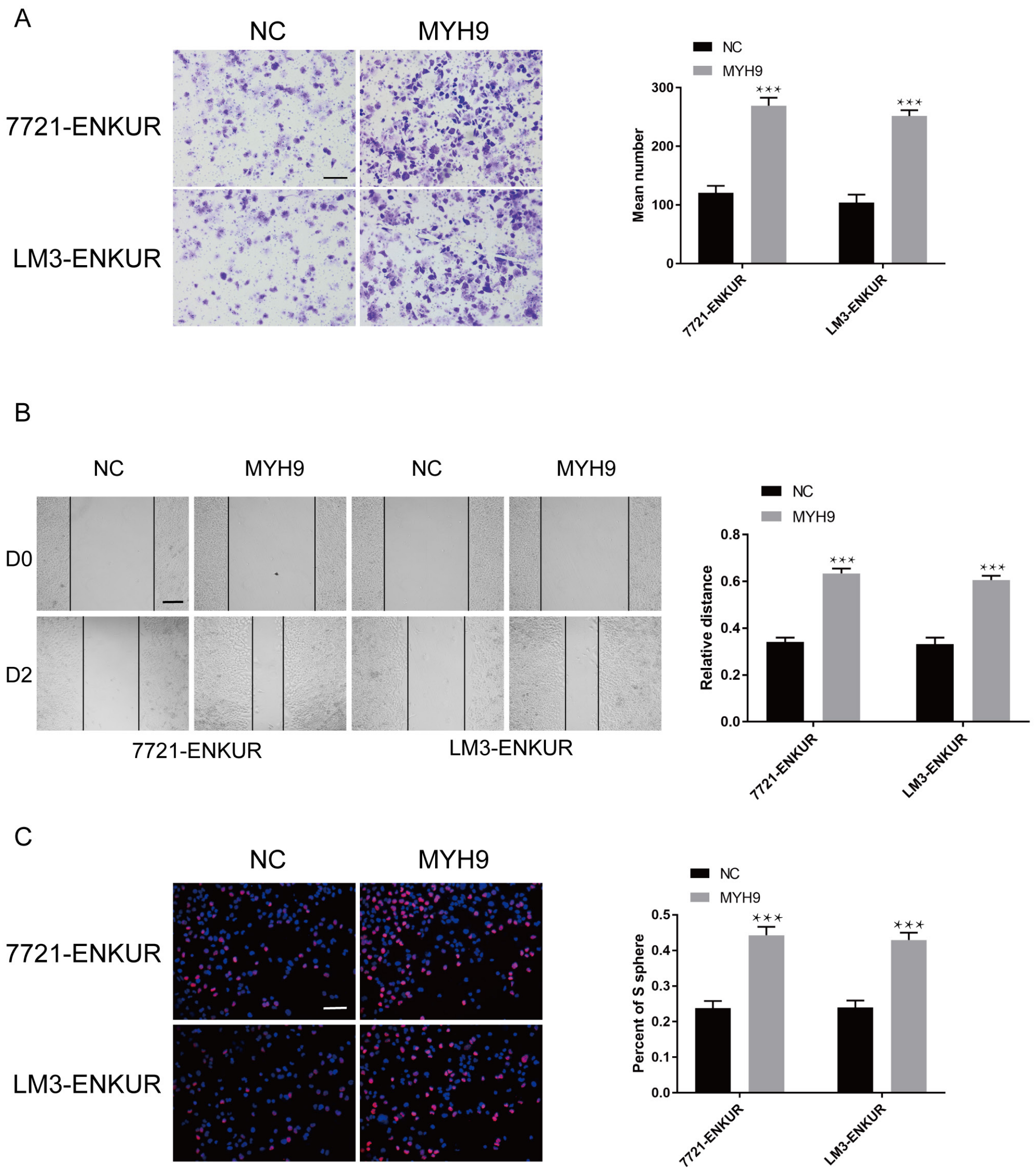

Figure S3. MYH9 reverses the inhibition of ENKUR.

(a-c) Boyden assay, wound healing assay and EDU assay showing the malignant behaviors after MYH9 treatment.

Supplementary figure 4

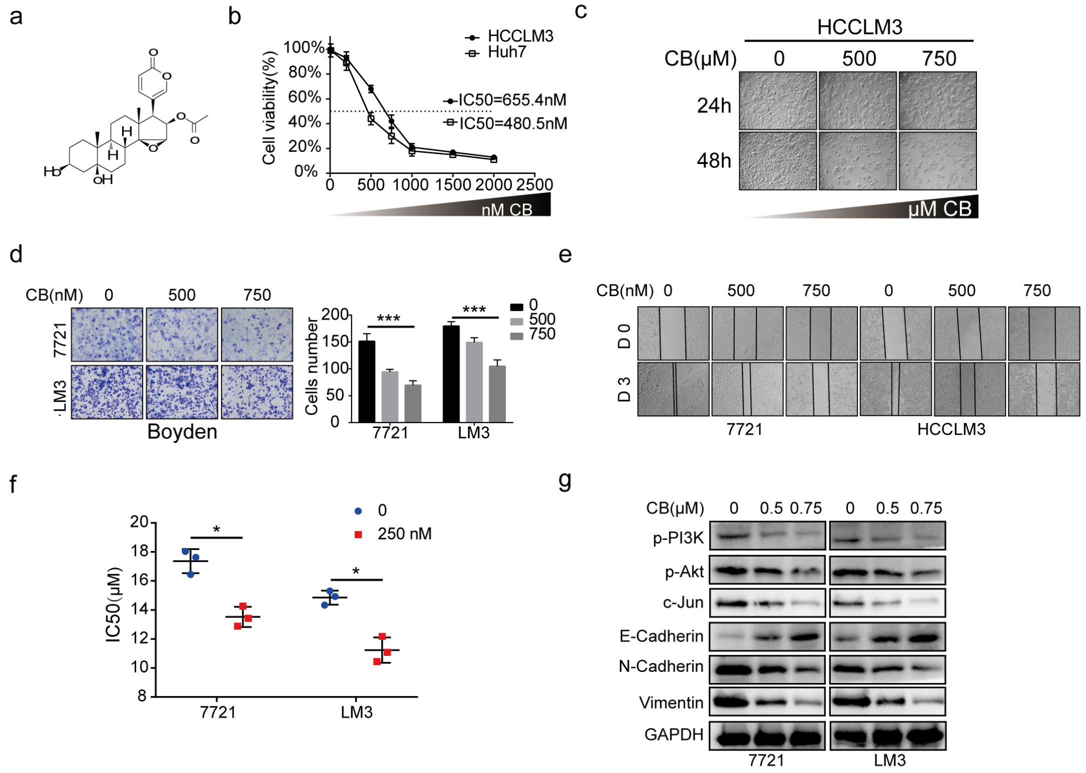

Figure S4. CB inhibits tumor proliferation, metastasis, and sorafenib resistance in HCC cells.

(a)Chemical structure formula of CB; (b) MTT assays showing the IC50 of CB in HCC cells; (c) MTT assays showing the proliferation of HCC cells after CB treatment; Transwell (d) and wound healing (e) assays showing tumor invasion and migration of HCC cells after CB treatment; (f) IC50 of sorafenib in HCC cells after CB treatment; (g) Western blot assays showing pPI3K, p-AKT, c-Jun, N-cadherin, E-cadherin, and vimentin expressions

Supplementary Table1: The sequences used in this study.

Name	Sequence
ENKUR-1	GGACTAGTTCCAAAGTACA
ENKUR-2	ACACGACATTGGCATAATT

Supplementary Table2: The primers used in this study.

Primers name		Sequence (5’-3’)
ENKUR	Forward	CCAGTTCAACCTCCCCCAAT
	Reverse	GGGGCCAGACCACATCAAAT
MYH9	Forward	AGTTTGTCTCGGAGCTGTGG
	Reverse	GGTTCGTGTTCCTCAGCGTA
GAPDH	Forward	GCACCGTCAAGGCTGAGAAC
	Reverse	TGGTGAAGACGCCAGTGGA
Chip-c-Jun-A	Forward	GGCTGAGGCAAGAGGATTT
	Reverse	GCCACTGTGTCTGGTCTTT
Chip-c-Jun-B	Forward	TAACACGTTCCTCCGAACAC
	Reverse	GGCTGATGGCACCTCTG

Supplementary Table 3: The sequences used in Electrophoretic mobility shift assay.

Gene	Type		Sequence(5'-3')
c-Jun	probes	wild type	ACACGCACGGTGGCTGAGGAAATCAGCT ACTTGAGCTATGATGATGCCACTACACT
	competitors	wild type	ACACGCACGGTGGCTGAGGAAATCAGCT ACTTGAGCTATGATGATGCCACTACACT
		Mutant 1	ACACGCACGGcttagtcttgggaCAGCTACTTGA GCTATGATGATGCCACTACACT
		Mutant 2	ACACGCACGGTGGCTGAGGAAATCAGCT ACTTGAGCTgctgctgctaagaTACACT
		Mutant 1+2	ACACGCACGGcttagtcttgggaCAGCTACTTGA GCTgctgctgctaagaTACACT
